# Coumarin-Based Photolabile Solid Support Facilitates Nonchromatographic Purification of RNA Oligonucleotides

**DOI:** 10.1021/acs.joc.5c01528

**Published:** 2025-11-10

**Authors:** Ian McClain, Hilal Dagci, Bhoomika Pandit, Maksim Royzen

**Affiliations:** 1084 University at Albany, Department of Chemistry, 1400 Washington Ave., Albany, New York 12222, United States

## Abstract

We describe the synthesis and application of a coumarin-based photocleavable anchor for solid-phase synthesis and nonchromatographic purification of RNA oligonucleotides. In contrast to standard nitroaryl anchors, which are cleaved with ultraviolet (UV) light, the described construct requires visible light (λ = 456 nm) for photocleavage. This is especially important for RNAs that contain a phosphorothioate backbone, which is not compatible with UV light. We optimized the synthesis and purification process using a model 20-nt poly-U RNA and subsequently applied it to structured and unstructured RNA oligonucleotides that contain both phosphodiester and phosphorothioate backbones. Lastly, we purified 103-nt sgRNA and checked its functional fidelity by CRISPR experiments.

## Graphical abstract

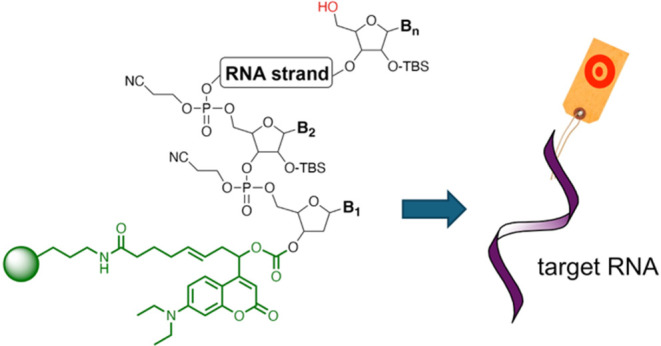

## Introduction

The rapid growth of biomedical RNA research created a strong need for innovation in chemical RNA synthesis. Such need is driven by the FDA approval of multiple siRNA-based drugs, including patisiran (2018), givosiran (2019), lumasiran (2020), and inclisiran (2022).^1^ The active ingredients of these drugs are currently manufactured using classical solid-phase synthetic procedures and purified by preparative HPLC. In recent years, we have witnessed a rapid development of CRISPR-Cas9-based research tools and therapeutics.^2^ The key ingredient of these technologies is single-guide RNA (sgRNA), which is about 100-nt long and contains multiple modifications that improve its *in vivo* stability.^3–5^ These sgRNAs are especially difficult to synthesize and purify using classical approaches due to their length and structural complexity.

Currently, HPLC is the most common approach to purify synthetic RNA oligonucleotides. Out of many different chromatographic methods, reverse-phase (RP) and anion-exchange chromatography are the most prevalent.^6^ The former is typically carried out using oligonucleotides protected with a highly hydrophobic dimethoxytrityl (DMT) group to further differentiate their polarity from many impurities that accumulate during solid-phase synthesis. The DMT group is subsequently chemically cleaved post-purification. On the other hand, anion-exchange chromatography leverages the anionic nature of multiple phosphate groups on the backbone of RNA to build strong attraction forces with the positively charged amine ligands on the solid support.^7^ A number of innovative approaches to facilitate chromatographic oligonucleotide purification have been reported. For example, scientists at Berry and Associates developed fluorous oligonucleotide tags to facilitate fluorous affinity chromatography.^8^ Also, DNA-affinity chromatography has been reported using DNA-modified silica chromatographic column.^9^ Despite considerable progress in terms of methods and modern instrumentation, long oligonucleotides remain difficult to purify chromatographically because of structural similarities between the target strand and the failure sequences. For RNAs that are over 100-nt, the target strand often has to be purified from a crude mixture containing more than 90% impurities.

A number of innovative nonchromatographic purification methods have emerged in recent years to address these challenges.^10^ For example, Fang and co-workers developed acrylated phosphoramidites for selective modification of either the target or failure sequences.^11,12^ Nonchromatographic purification was accomplished by polymerization, which facilitates facile differentiation of target and failure sequences. The method has been applied to isolate a 1728-nt-long oligonucleotide.^13^ Bergstrom reported a purification approach that entails biotinylation of the 5′-end of synthetic RNAs.^14,15^ Postsynthetically, the target strands were captured with NeutrAvidin-coated microspheres. The Minakawa group described a “catch and release” oligonucleotide purification strategy that combined strain-promoted alkyne–azide cycloaddition and photocleavage.^16^ Our lab also reported a nonchromatographic purification procedure, which utilizes inverse electron demand Diels–Alder chemistry between *trans*-cyclooctene (TCO) and tetrazine (Tz), illustrated in Scheme 1.^17^

**Scheme 1:**
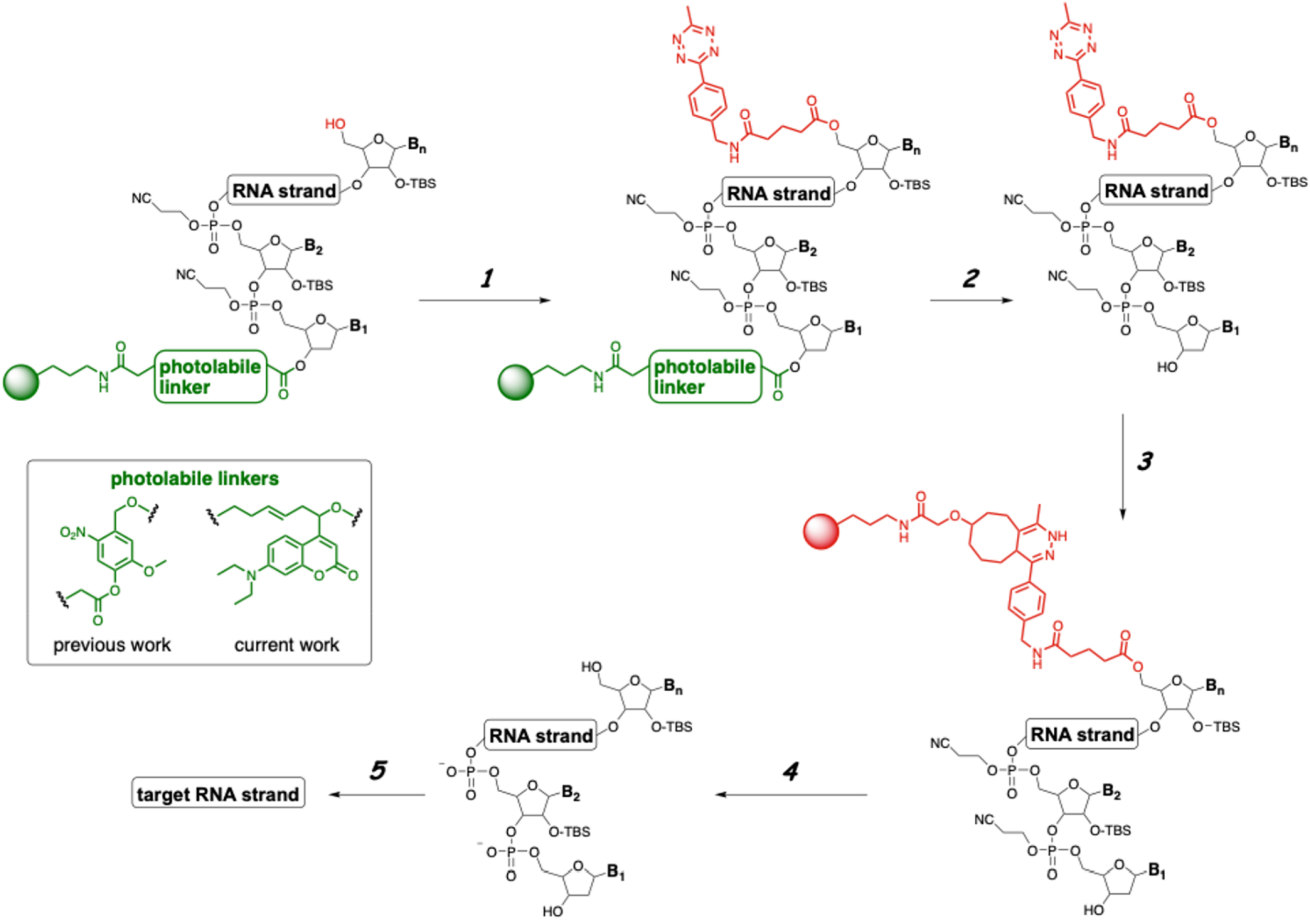
Five-Step Procedure for Nonchromatographic Purification of Synthetic RNA Oligonucleotides^a^

a (1) Tagging of the target strand with Tz anhydride; (2) photocleavage; (3) capture of the target strand with CPG-TCO; (4) deprotection; (5) desilylation and ethanol precipitation.

Our procedure takes advantage of the free 5′–OH group on the target strand, and we can selectively tag the target with Tz (step 1). The key element of this process is a photolabile linker, which facilitates cleavage from the solid support in step 2, while preserving all the protecting groups on the RNA. The target strand can subsequently be captured using solid support-immobilized TCO and purified from the failure strands, which will be dissolved and washed away in the supernatant (step 3). The target RNA strand is isolated using standard cleavage, deprotection, desilylation, and ethanol precipitation steps. In our original report, we utilized an o-nitroaryl photocleavable linker developed by Greenberg et al.^18^ The photocleavage step entailed exposure to 350 nm of light for 2 h. In the current work, we developed a coumarin-based photolabile linker that requires irradiation with visible light (456 nm) for a shorter period of time. This linker is particularly useful for RNAs that contain groups that are sensitive to UV light, such as the phosphorothioate backbone.

## Results and Discussion

Coumarin-based probes have been utilized extensively for photocaging of proteins and nucleic acids.^19–22^ Our aim was to synthesize a heterobifunctional coumarin-based photolabile linker suitable for solid-phase RNA synthesis. One end of the linker will be attached to the first oligonucleotide subunit. As a proof-of-concept, we chose thymidine as the first subunit. The other end is immobilized on the CPG solid support. To achieve that, we developed a synthetic procedure shown in Scheme 2. The synthesis commenced with the previously reported compounds **1** and **2** that were prepared using the reported methods.^23,24^ Coupling of **1** and **2** in the presence of DMAP produced compound **3**. The 2,4,5-trichlorophenyl ester moiety, needed for the attachment to CPG, was installed using a cross-metathesis reaction between compounds **3** and **4**. Several second-generation Grubbs catalysts were screened for this step, and the best yield was obtained using Hoveyda–Grubbs catalyst M731. Finally, compound **5** was immobilized on a CPG1000A solid support. CPG loading was determined to be 15 μmol/g by cleaving the DMT group with trichloroacetic acid and measuring absorbance at 504 nm.

**Scheme 2:**
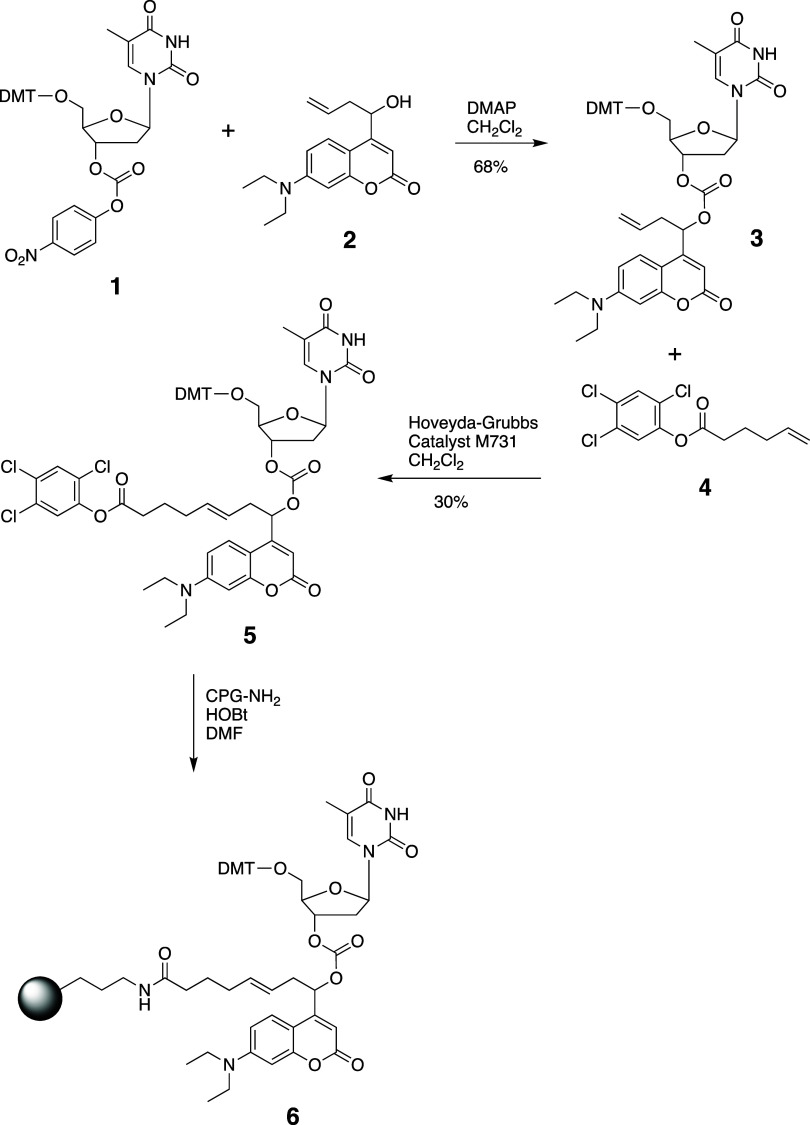
Synthesis of the Coumarin-Based Photolabile Linker Attached to CPG

To optimize the photocleavage step (Scheme 1, step 2), we synthesized a model 20-nt poly-U RNA strand, termed **RNA1**. While on support, the oligonucleotide was tagged with the freshly prepared Tz anhydride following the protocol described in our previous work (Scheme 1, step 1).^17^ The photocleavage was carried out using a Kessil lamp (PR160L) at 75% power. We tested 15, 30, and 60 min of irradiation, which was followed by deprotection, desilylation, and EtOH precipitation. The HPLC analysis of isolated RNAs is shown in Figure S1A. The highest isolated yield of RNA was obtained after 30 min of photocleavage. As illustrated in Figure S1B, relative to the AMA treatment, 30 min of photoirradiation enabled cleavage of 93% of **RNA1**. After optimization, the photocleavage step was repeated using a fresh sample and the cleaved oligonucleotide was captured by TCO-modified CPG beads using the previously described procedure (Scheme 1, step 3).^25^ During this step, the failure strands lacking the Tz tag were removed by filtration. The target **RNA1** was obtained upon treatment with a 1:1 aqueous methylamine-ammonia solution (AMA), followed by desilylation with triethylamine trihydrofluoride and EtOH precipitation. Figure 1A shows the HPLC analysis of the isolated **RNA1** strand. Its identity was verified using a reference mixture of 17, 18, 19, and 20-nt poly-U RNA strands that were chromatographed using the same HPLC conditions (Figure 1B). Purity of isolated **RNA1** was determined to be 86.2%. The isolated **RNA1** strand was also analyzed by ESI-MS. The deconvoluted mass spectrum, shown in Figure 1C, matched the calculated *m*/*z* value of 6056.52. Based on the nanodrop measurements, the isolated yield of the target RNA was 30%.

**Figure 1:**
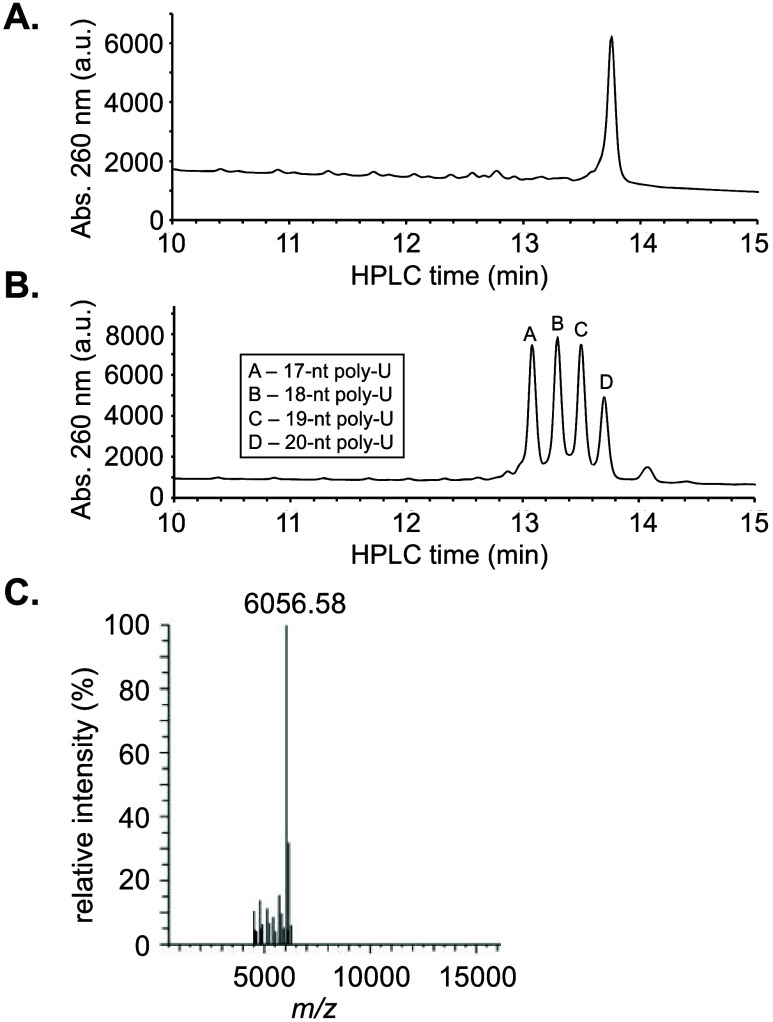
(A) HPLC analysis of the isolated 20-nt poly-U RNA oligonucleotide. (B) Reference HPLC spectrum of a mixture containing 17, 18, 19, and 20-nt poly-U RNA strands. (C) Deconvoluted ESI-MS analysis of the isolated 20-nt poly-U RNA oligonucleotides.

To confirm that our approach is general, we applied the nonchromatographic RNA purification procedure toward the isolation of 20-nt stem-loop RNA (**RNA2**) with the sequence: 5′-ACCUGGCUUUCACCCAGGUT-3′ and a nonstructured 20-nt RNA (**RNA3**) with the sequence: 5′-GAUCCUGCCGACUACGCCAT-3′. Both RNAs were synthesized using **6** and were purified using the process described in Scheme 1. Figure 2A,B shows the HPLC spectra of purified **RNA2** and **RNA3**. Both spectra show dominant target peaks with trace numbers of failure sequences. Purities of the isolated **RNA2** and **RNA3** were determined to be 84.6 and 86.7%, respectively. The isolated **RNA2** and **RNA3** strands were also analyzed by ESI-MS. The deconvoluted mass spectra are shown in Figure 2C,D. The observed *m*/*z* value of 6274.88 for **RNA2** matched the calculated value of 6274.83. The observed *m*/*z* value of 6296.91 for **RNA3** matched the calculated value of 6296.89. Based on the nanodrop measurements, the isolated yields of **RNA2** and **RNA3** were 17 and 18%, respectively.

**Figure 2:**
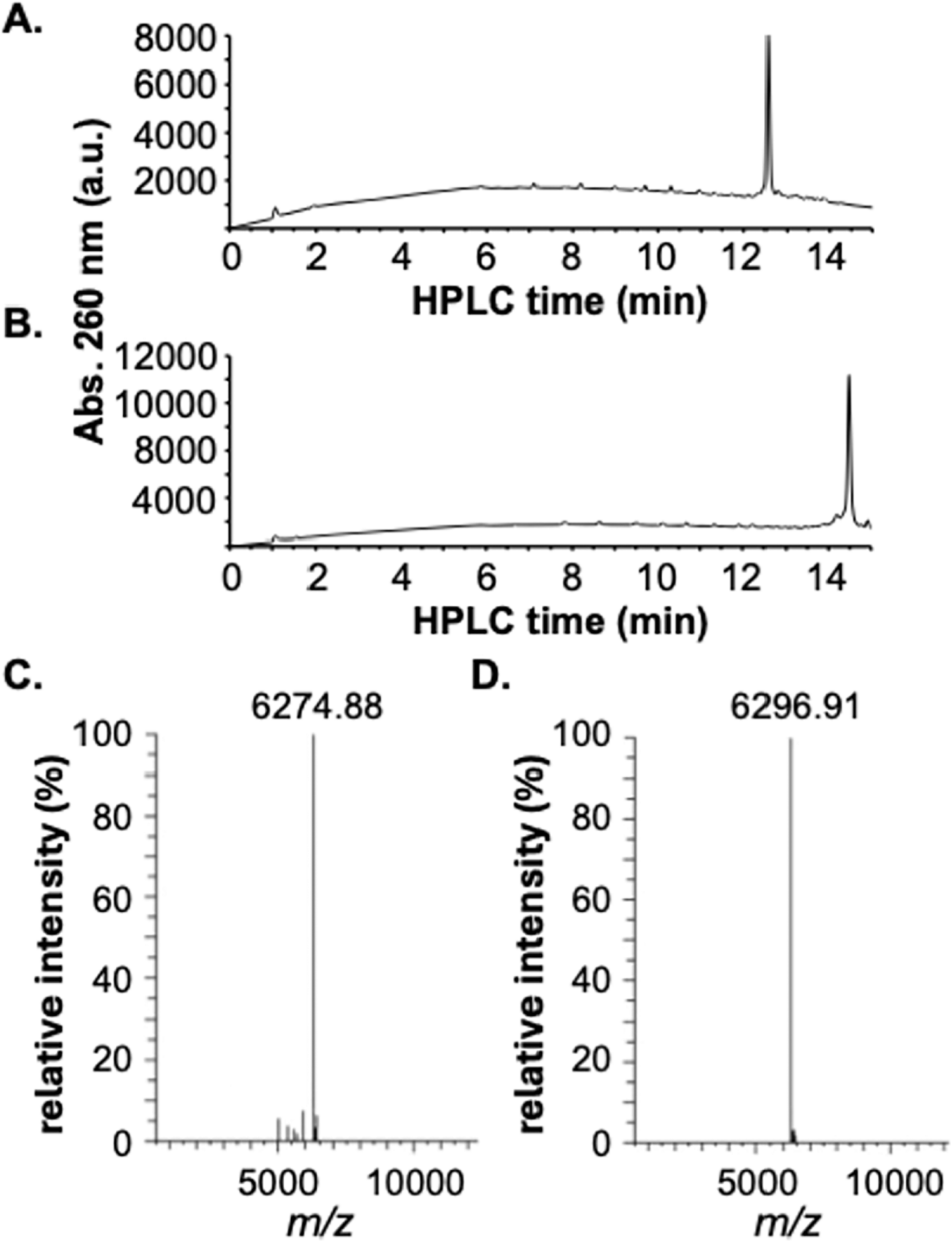
(A) HPLC analysis of the isolated **RNA2** oligonucleotide. (B) HPLC analysis of the isolated **RNA3** oligonucleotide. (C) Deconvoluted ESI-MS analysis of the isolated **RNA2** oligonucleotide. (D) Deconvoluted ESI-MS analysis of the isolated **RNA3** oligonucleotide.

An important advantage of the coumarin-based photolabile linker is that it is much more compatible with RNAs containing a phosphorothioate backbone. Prolonged exposure of the latter to 350 nm light can potentially cause photo-cross-linking, thus damaging the RNA’s structure and functions. Meanwhile, the phosphorothioate backbone is an important feature of many therapeutic RNAs known for enhancing *in vivo* stability against nuclease degradation.^26^ We tested our nonchromatographic RNA purification procedure toward the isolation of **RNA4** and **RNA5** that have the same sequences as **RNA2** and **RNA3** but contain a phosphorothioate backbone. Both RNAs were synthesized using **6** and were purified using the process described in Scheme 1. Figure 3A,B shows the HPLC spectra of purified **RNA4** and **RNA5**, respectively. Both spectra show dominant target peaks with trace amounts of failure sequences. Purities of isolated **RNA4** and **RNA5** were determined to be 97.4 and 97.2%, respectively. The isolated **RNA4** and **RNA5** strands were also analyzed by ESI-MS. The deconvoluted mass spectra are shown in Figure 3C,D. The observed *m*/*z* value of 6582.9 for **RNA4** perfectly matched the calculated one. The observed *m*/*z* value of 6604.9 for **RNA5** matched the calculated value of 6605.0. Based on the nanodrop measurements, the isolated yields of **RNA4** and **RNA5** were 29 and 26%, respectively.

**Figure 3:**
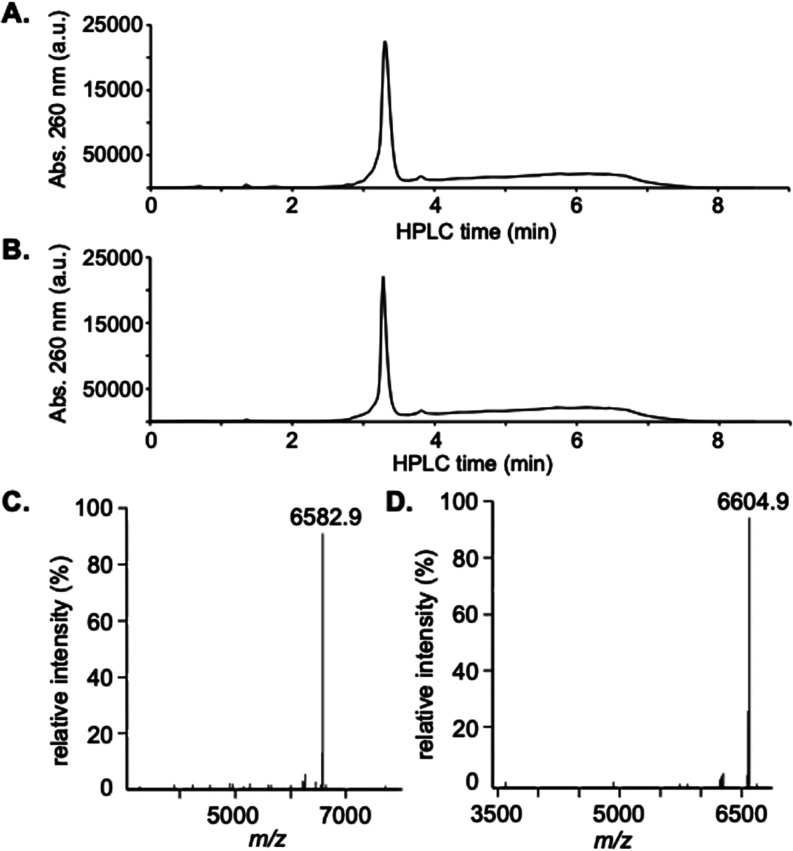
(A) HPLC analysis of the isolated **RNA4** oligonucleotide. (B.) HPLC analysis of the isolated **RNA5** oligonucleotide. (C) Deconvoluted ESI-MS analysis of the isolated **RNA4** oligonucleotide. (D) Deconvoluted ESI-MS analysis of the isolated **RNA5** oligonucleotide.

To further illustrate the advantages of the coumarin-based photolabile linker, we synthesized **RNA4** and **RNA5** using previously reported nitroaryl-based photolabile solid support, shown in Scheme 1.^17,25^ Subsequently, we carried out our previously reported nonchromatographic purification which commenced with irradiation using 350 nm light for 2h. Upon completion of the process, the isolated materials were analyzed by HPLC, as shown in Figure S2. The spectra are indicative of complex mixtures, which likely resulted due to susceptibility of the phosphorothioate backbone to prolonged exposure to UV light.

To show the practicality of our method, we synthesized a 103-nt-long **sgRNA** used for CRISPR experiments. The **sgRNA** was synthesized using **6** and was purified nonchromatographically, using the process described in Scheme 1. The isolated yield of **sgRNA** was calculated to be 29%. It is difficult to assess the purity of **sgRNA** using HPLC, because long RNAs can fold into a variety of different three-dimensional structures that will have different retention times. In our previous work, we analyzed the same 103-nt sgRNA using the recently reported HPLC procedure which utilizes high pH conditions to suppress RNA folding.^27^ Instead, we assessed the functional fidelity of experimentally purified **sgRNA** by performing CRISPR experiments. We compared the DNA-cutting efficiency of the experimentally purified **sgRNA** and standard **sgRNA** that was purified by preparative PAGE.

The ability of experimentally purified **sgRNA** to carry out Cas9-assisted cleavage of dsDNA was analyzed by agarose gel electrophoresis, as shown in Figure 4. Lane 1 is a DNA ladder. Lane 2 contains the linearized pBR322 plasmid. Lane 3 shows the activity of the standard **sgRNA** that was purified by preparative PAGE. After 16 h of treatment with the standard **sgRNA** and Cas9, we observed 97% DNA-cutting efficiency. The experimentally purified **sgRNA** shows similar CRISPR-Cas9 activity (Lane 4). The experiments were performed in triplicate. After 16 h of treatment, an average of 95% DNA-cutting efficiency was achieved. These results illustrate that the nonchromatographic RNA purification procedure facilitates the isolation of long RNAs that function on par with the ones purified by preparative PAGE.

**Figure 4:**
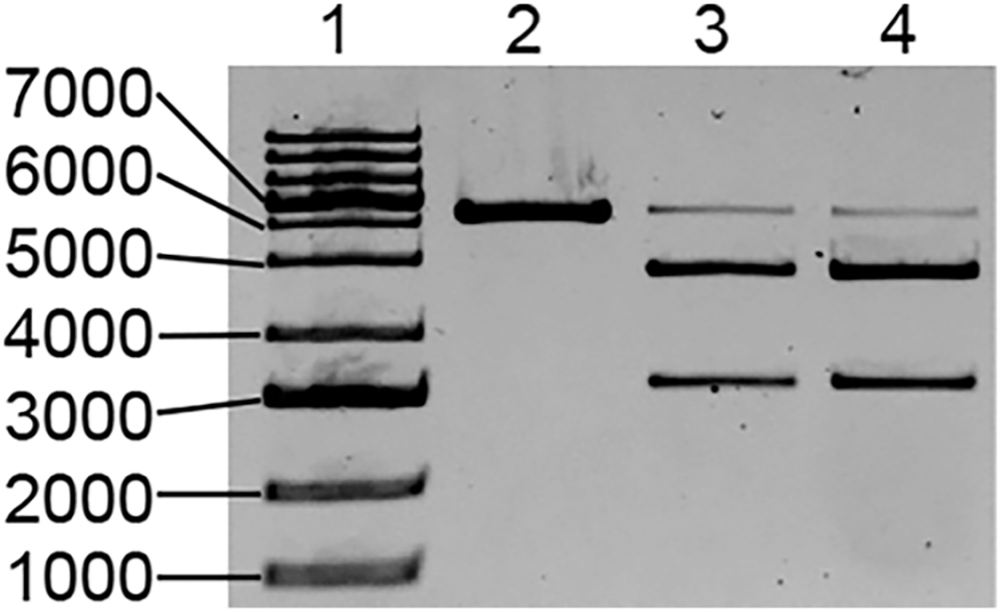
Analysis of CRISPR-Cas9 experiments using agarose gel electrophoresis. *Lane 1:* DNA ladder; *Lane 2:* linearized pBR322 plasmid; *Lane 3:* linearized pBR322 plasmid treated with standard **sgRNA** Cas9 for 16 h; and *Lane 4:* linearized pBR322 plasmid treated with the experimentally purified **sgRNA** Cas9 for 16 h.

## Conclusion

In conclusion, this report describes a new coumarin-based photolabile anchor that was utilized for the solid-phase synthesis of RNA oligonucleotides. Photocleavage was carried out using 456 nm light, which is compatible with RNAs containing phosphodiester and phosphorothioate backbones. Synthetic RNAs were isolated using a nonchromatographic purification process that we developed in our prior work.^17,25^ The photocleavage step was optimized using a model 20-nt poly-U oligonucleotide. Subsequently, the nonchromatographic purification procedure was applied to more complex RNAs, including 100-nt sgRNA for CRISPR experiments. In comparison to the standard RNA purification procedures using either RP-HPLC or anion-exchange chromatography, our process is faster and does not require expensive instrumentation and columns. By contrast, chromatographic purification typically entails lengthy optimization and preparation. However, it can facilitate the isolation of oligonucleotides in higher purity. In comparison to our previous work, we did not achieve significant improvement in terms of isolated yields. The main advantage of the new coumarin-based photolabile anchor is that it is more compatible with RNAs containing a phosphorothioate backbone. We have shown that the latter is stable to 456 nm light but sensitive to prolonged irradiation with UV light. The reported purification process, however, has a number of limitations in its current form. The process is not compatible with RNAs containing modifications at the 5′-end, such as a phosphate group. Also, the isolated oligonucleotides were not pure RNAs because their first nucleotide was deoxythymidine. In the future, we plan to adopt the reported synthetic procedures to make a uridine analog of compound **5**. We also plan to further optimize the process to obtain better isolated yields. The experiments shown in Figure S1 indicate that the photocleavage yields are comparable to those of the AMA treatment. Therefore, there are inefficiencies in other steps of the purification process, such as tagging or capture steps. We plan to improve them in our future work using more reactive Tz and TCO compounds,^28,29^ as well as alternative solid support materials to capture the photocleaved RNA.^30^ Furthermore, we plan to work on improving the purity of nonchromatographically isolated RNA. This will entail modification of the capping step of the solid-phase synthesis to ensure that all but 5′-hydroxyl groups are protected. To achieve that, we plan to explore alternative capping reagents. Our ultimate goal is for the coumarin-based photolabile anchor and the nonchromatographic purification process to be useful for the synthesis of therapeutic RNAs for various biomedical applications.

## Experimental Section

### General Information

The reagents and solvents used for the synthesis of compounds **1**–**6** were purchased from Sigma-Aldrich, Acros Organic, and Fisher Scientific. The organic solvents used for flash chromatography were purchased from Greenfield Global. For gel electrophoresis, 10× Tris/Borate/EDTA (TBE) buffer was purchased from Fisher Scientific and used with proper dilution. 30% Arcylamide/Bis-arcylamide solution (29:1) was purchased from Bio-Rad Laboratories, Inc. Chromatographic purifications of synthetic materials were conducted using SiliaSphere spherical silica gel with an average particle and pore size of 5 μm and 60 Å, respectively (Silicycle Inc., QC, Canada). Thin-layer chromatography (TLC) was performed on SiliaPlate silica gel TLC plates with 250 μm thickness (Silicycle Inc., QC, Canada). Preparative TLC was performed using SiliaPlate silica gel TLC plates with 1000 μm thickness. ^1^H and ^13^C NMR spectroscopy was performed on a Bruker NMR instrument at 500 MHz (^1^H) and 126 MHz (^13^C). All ^13^C NMR spectra were proton decoupled. High-resolution ESI-MS spectra were acquired using an Agilent Technologies 6530 Q-TOF instrument. The RNA and DNA samples were analyzed on a Thermo Fisher Scientific (West Palm Beach, CA) LTQ Orbitrap Velos Mass spectrometer using quartz capillary emitters. To facilitate spray optimization, 10% isopropyl alcohol was added to each sample prior to MS analysis. Purification of all synthetic oligonucleotides was characterized by denaturing urea polyacrylamide gel electrophoresis (PAGE) (15 wt %, Acrylamide:Bis-acrylamine = 29:1, 1× TBE buffer). Gels were then stained with Ethidium Bromide (EthBr, 1 μg/mL) and visualized with a ChemDoc Imaging System. Low-range ssRNA ladder (New England Biolabs cat# N0364S) was used as shown in Figure 4.

All oligonucleotide solid-phase syntheses were performed on a 0.5 μmol scale using the Oligo-800 synthesizer (Azco Biotech, Oceanside, CA, USA). Solid-phase syntheses were performed on a control-pore glass (CPG 1000) purchased from Glen Research (Sterling, VA, USA). Other oligonucleotide solid-phase synthesis reagents were obtained from ChemGenes Corporation (Wilmington, MA, USA). Phosphoramidites (TBDMS as the 2′-OH protecting group): rA was N-Bz protected, rC was N-Ac protected, and rG was N-iBu protected. The coupling step was done using 5-ethylthio-1H-tetrazole solution (0.25 M) in acetonitrile for 12 min. The 5′-detritylation step was done using 3% trichloroacetic acid in CH_2_Cl_2_. Oxidation was done using I_2_ (0.02 M) in a THF/pyridine/H_2_O solution. CPG modifications were carried out using native amino lcaa CPG 1000 Å, purchased from ChemGenes (Wilmington, MA, USA), Cat.# N-5100-10.

#### Standard Oligonucleotide Purification Procedure

Upon completion of the solid-phase synthesis, the solid support was dried with a Speed-Vac concentrator. The oligonucleotides were cleaved from the solid support and were fully deprotected with an AMA solution (a 1:1 aqueous solution of methylamine and concentrated ammonium hydroxide) at 65 °C for 45 min. The solution was evaporated to dryness by using a Speed-Vac concentrator. DNA samples were redissolved in water and characterized by PAGE and nanodrop. For RNA samples, the solid was dissolved in DMSO (100 μL) and was desilylated using a solution of Et_3_N·3HF at 65 °C for 2.5 h. After cooling to room temperature, the RNA was precipitated with 3 M sodium acetate (25 μL) and ethanol (1 mL). The solution was cooled to −20 °C overnight before the RNA was recovered by centrifugation and finally dried under a vacuum. Target oligonucleotide strands were purified by preparative gel electrophoresis.

Preparative gel purification of RNA was carried out using denaturing urea polyacrylamide gel electrophoresis (PAGE) (15 wt %, Acrylamide:Bis-acrylamine = 29:1, 1× TBE buffer). Target RNA was visualized with UV light (254 nm). The band containing the target RNA was cut with a clean razor blade and chopped into fine particles. Gel stabs were transferred to a 1.5 mL Eppendorf tube, and RNA was eluted with 800 μL of elution buffer (500 mM ammonium acetate, 10 mM magnesium acetate, 2 mM EDTA) on a rotary shaker for 24 h at rt. After elution, the gel fragments were spun down, and the supernatant was transferred to another Eppendorf tube. The supernatant solution containing RNA was washed several times with an equal volume of n-butanol. The wash step was repeated several times until the volume of the lower aqueous portion was convenient for RNA precipitation. The RNA was precipitated after the addition of ethanol (1 mL) and cooling at −20 °C for 18 h. The purified RNA was pelleted by centrifugation and resuspended in Milli-Q water for characterization.

HPLC analysis of **RNA1**, **RNA2**, and **RNA3** was performed on a Shimadzu LC-20 Instrument, equipped with a DNAPac PA200 BioLC analytical column (Thermo Scientific). Running **buffer A** contained 20 mM TRIS Base, acidified with conc. HCl to a final pH of 8. Running **buffer B** contained 20 mM TRIS Base and 1.25 M NaCl, pH 8. The gradient (0–90% **buffer B**) employed at 1.2 mL per min flow rate was utilized with an oven temperature of 60 °C.

HPLC analysis of the **RNA4** and **RNA5**, containing phosphorothioate backbone RNA was performed on Shimadzu Nexera 40 Instrument, equipped with Kinetex 2.6 μm XB-C18 100 Å column (Phenomenex) using previously reported methods.^31,32^ Running **buffer A** contained 14.3 mM triethylamine and 114 mM hexafluoroisopropanol dissolved in water containing 2.5% v/v CH_3_OH. Running **buffer B** contained 14.3 mM triethylamine and 114 mM hexafluoroisopropanol dissolved in a 3:2 solution of CH_3_OH:H_2_O (v/v). The gradient (0–80% **buffer B**) employed at 0.6 mL per min flow rate was utilized with a Kinetex 2.6 μm XB-C18 100 Å column and an oven temperature of 65 °C.

#### HPLC-Free Oligonucleotide Purification Procedure Described in This Work

Our experimental procedure utilized a CPG-based solid support that was functionalized with a coumarin-based photolabile group. Oligonucleotides were synthesized by the standard solid-phase synthesis described above. Upon completion, the solid support (20–30 mg) was dried with a Speed-Vac concentrator. On support tagging with a freshly prepared Tz anhydride was carried out using conditions described in our previous work.^17^ Successful capping with Tz resulted in the CPG beads turning purple. Oligonucleotides were subsequently cleaved from the solid support using a 456 nm light. Cleavage was done using a Kessil PR160L LED PhotoReaction Lighting 456 nm (Kessil Lighting Richmond, CA, USA). CPG beads were placed in a quartz cuvette (Starna Scientific, Atascadero, CA, USA; cat.# GL14/S), a 1:1 solution of CH_3_CN:H_2_O (2 mL) was added and the resulting suspension was degassed with N_2_ for 30 min. The photocleavage was carried out for 30 min under a N_2_ atmosphere. The supernatant solution containing cleaved oligonucleotides was separated from CPG beads and concentrated to ∼1 mL in a Speed-Vac concentrator. The supernatant solution was treated with TCO-functionalized CPG beads to capture the target strands. TCO–CPG beads (100 mg) were added to the supernatant solution and placed in a thermoshaker at 37 °C for 2 h. Supernatant solution was removed, and the captured RNA beads were washed with CH_3_CN (200 μL), followed by H_2_O (200 μL). The RNA beads were dried using Speed-Vac concentrator. Captured target oligonucleotides were cleaved from the CPG beads and were fully deprotected with AMA solution using the standard conditions. The solution was evaporated to dryness using a Speed-Vac concentrator. For the synthetic RNA samples, the solid was dissolved in DMSO (100 μL) and was desilylated using a solution of Et_3_N·3HF at 65 °C for 2.5 h. After cooling to rt, the RNA was precipitated with 3 M sodium acetate (25 μL) and ethanol (1 mL). The solution was cooled to −20 °C overnight before the RNA was recovered by centrifugation and finally dried under vacuum.

#### Oligonucleotide Strands Used in This Work

**RNA1**: 5′-UUU UUU UUU UUU UUU UUU UT-3′

**RNA2**: 5′-ACC UGG CUU UCA CCC AGG UT-3′

**RNA3**: 5′-GAU CCU GCC GAC UAC GCC AT −3′

**RNA4**: 5′-ACC UGG CUU UCA CCC AGG UT-3′ PS backbone

**RNA5**: 5′-GAU CCU GCC GAC UAC GCC AT −3′ PS backbone

**103-nt sgRNA**:

5′-GGGCGCUUGUUUCGGCGUGGGUAGUUUUAGAGCUAGACAUAGCAAGUU

AAAAUA AGGCUAGUCCGUUAUCAACUUGAA AAAGUGGCACCGAGUCGGUGCUUUT-3′

#### Cas9 In Vitro Cleavage Assay

pBR322 plasmid DNA (0.35 μM, 1.13 μL, NEB, N3033S) was diluted with water (16.87 μL) and NEB buffer 3.1 (10×, 2 μL). The plasmid was linearized directly prior to CRISPR experiments using PvuII (10 U/μL, 1 μL, NEB, R0151S) for 1 h at 37 °C. In an analogous fashion, eGFP-N1 plasmid was linearized with DraIII-HF (10 U/μL, 1 μL, NEB, R3510L). For the Cas9-mediated DNA cleavage assay, sgRNA (300 nM, 5 μL), Cas9 (1 μM, 0.3 μL, NEB, M0386S), Cas9 buffer (10×, 1 μL, NEB), linearized plasmid (20 nM, 1.5 μL), and H2O (2.2 μL) were mixed together (final volume = 10 μL) and incubated for either 1 or 16 h at 37 °C. CRISPR experiments were terminated by the addition of proteinase K (20 mg/mL, 0.5 μL) for 1 h at 37 °C. The reaction (1 μL) was mixed with blue loading buffer (6×, 2 μL, NEB, B7703S) and loaded on 1% agarose stained with ethidium bromide (1× TBE running buffer). The agarose gel images were processed using BioRad Image Lab software (version 6.0), which is part of the BioRad Chemidoc MP Imaging System. DNA-cutting efficiency in Figure 4 was determined by quantifying the intensities of the top band in lanes 3,4, and 5 relative to the intensity of the linearized plasmid in lane 2. The reported DNA-cutting efficiency values are averages of triplicate experiments.

##### (2R,3S,5R)-2-((Bis­(4-methoxyphenyl)­(phenyl)­methoxy)­methyl)-5-(5-methyl-2,4-dioxo-3,4-dihydropyrimidin-1­(2H)-yl)­tetrahydrofuran-3-yl (1-(7-(Diethylamino)-2-oxo-2H-chromen-4-yl)­but-3-en-1-yl) Carbonate (**3**)

Compound **2** (500 mg, 0.705 mmol) and DMAP (130 mg, 1.058 mmol) were dissolved in anhydrous CH_2_Cl_2_ (50 mL). Coumarin **1** (430 mg, 0.846 mmol) was added. The reaction flask was wrapped in foil to prevent ambient light exposure, and the reaction mixture was stirred at rt for 24 h. The reaction mixture was diluted with CH_2_Cl_2_ (100 mL) and washed with sodium bicarbonate (100 mL). The organic phase was then dried with anhydrous Na_2_SO_4_ and concentrated under reduced pressure. The product was purified by flash chromatography using a gradient of MeOH in CH_2_Cl_2_ (5–10%), affording an oily yellow-brown solid. Yield = 0.380 g (68%)

^1^H NMR (500 MHz, CDCl_3_) δ 8.86 (d, *J* = 20.9 Hz, 1H), 7.61 (dd, *J* = 28.2, 1.4 Hz, 1H), 7.47–7.34 (m, 3H), 7.32–7.24 (m, 7H), 6.84 (ddd, *J* = 8.8, 4.1, 1.5 Hz, 4H), 6.63 (ddd, *J* = 9.2, 3.9, 2.7 Hz, 1H), 6.56–6.43 (m, 2H), 6.10 (d, *J* = 11.0 Hz, 1H), 5.88–5.71 (m, 2H), 5.38–5.33 (m, 1H), 5.24–5.12 (m, 2H), 4.22 (d, *J* = 2.1 Hz, 1H), 3.85–3.74 (m, 7H), 3.56–3.37 (m, 6H), 2.79–2.64 (m, 2H), 2.64–2.39 (m, 2H), 1.38 (t, *J* = 1.5 Hz, 3H), 1.23 (td, *J* = 7.1, 3.9 Hz, 6H).

^13^C­{^1^H} NMR (126 MHz, CDCl_3_): δ 163.7, 161.8, 158.8, 156.7, 153.7, 150.7, 150.4, 144.1, 135.4, 135.1, 131.6, 130.1, 128.1, 127.3, 124.9, 119.5, 113.4, 111.7, 108.8, 105.7, 98.0, 87.3, 84.4, 84.0, 83.6, 79.6, 75.3, 75.1, 63.7, 55.3, 44.8, 39.0, 38.1, 37.6, 12.5, 11.6. HRMS (ESI) Calc’d for C_49_H_52_N_3_O_11_ [M + H]^+^ = 858.3596; found = 858.3579

##### 2,4,5-Trichlorophenyl (E)-8-(((((2R,3S,5R)-2-((bis­(4-methoxyphenyl)­(phenyl)­methoxy)­methyl)-5-(5-methyl-2,4-dioxo-3,4-dihydropyrimidin-1­(2H)-yl)­tetrahydrofuran-3-yl)­oxy)­carbonyl)­oxy)-8-(7-(diethylamino)-2-oxo-2H-chromen-4-yl)­oct-5-enoate (**5**)

Compound **3** (230 mg, 0.268 mmol) was dissolved in anhydrous CH_2_Cl_2_ (40 mL) inside a foil-wrapped flask. Hoveyda–Grubbs Catalyst M731 (22 mg, 10 mol %) was added. 1-(2,4,5-Trichlorophenol)-hexenoic acid (786 mg, 2.684 mmol) was added in 5 equal portions every hour. The reaction mixture was then stirred overnight at rt. The product was purified by flash chromatography using a gradient of EtOAc in heptane (20–80% yield), affording a pale yellow solid. Yield = 0.090 g (30%).

^1^H NMR (500 MHz, CDCl_3_) δ 8.13 (d, *J* = 4.8 Hz, 1H), 7.65–7.50 (m, 2H), 7.47–7.33 (m, 3H), 7.33–7.18 (m, 8H), 6.84 (ddt, *J* = 9.0, 3.3, 1.6 Hz, 4H), 6.62 (dt, *J* = 9.1, 3.1 Hz, 1H), 6.57–6.43 (m, 2H), 6.08 (d, *J* = 10.1 Hz, 1H), 5.77 (dt, *J* = 23.6, 6.2 Hz, 1H), 5.64–5.44 (m, 2H), 5.34 (dt, *J* = 10.7, 5.5 Hz, 1H), 4.25–4.17 (s, 1H), 3.79 (t, *J* = 2.2 Hz, 6H), 3.56–3.35 (m, 6H), 2.75–2.39 (m, 6H), 2.16 (dq, *J* = 14.5, 6.5 Hz, 2H), 1.82 (dq, *J* = 14.3, 7.0 Hz, 2H), 1.62 (s, 6H), 1.40–1.31 (m, 3H), 1.30–1.16 (m, 6H).

^13^C­{^1^H} NMR (126 MHz, CDCl_3_) δ: 170.5, 163.3, 161.8, 158.8, 156.6, 153.7, 153.5, 152.9, 152.8, 150.7, 150.2, 145.8, 144.1, 135.1, 134.1, 131.0, 130.0, 128.0, 127.2, 126.1, 125.4, 124.5, 113.4, 111.6, 108.8, 105.6, 98.0, 87.3, 84.3, 83.6, 79.6, 76.7, 75.3, 63.8, 63.7, 55.2, 44.7, 38.1, 33.9, 33.0, 32.9, 24.1, 12.4, 11.5.

HRMS (ESI) Calc’d for C_59_H_58_Cl_3_N_3_O_13_ [M + H]^+^ = 1122.3108; Found = 1122.3113

## Supplementary Material



## Data Availability

The data underlying this study are available in the published article and in its Supporting Information.

## Notes

NMR spectra of the synthesized compounds (PDF)

## References

[1] Egli M., Manoharan M. (2023). Chemistry, Structure and Function of Approved Oligonucleotide
Therapeutics. Nucleic Acids Res..

[2] Li T., Yang Y., Qi H., Cui W., Zhang L., Fu X., He X., Liu M., Li P., Yu T. (2023). CRISPR/Cas9
Therapeutics: Progress and Prospects. Signal
Transduct. Target Ther..

[3] O’Reilly D., Kartje Z. J., Ageely E. A., Malek-Adamian E., Habibian M., Schofield A., Barkau C. L., Rohilla K. J., Derossett L. B., Weigle A. T., Damha M. J., Gagnon K. T. (2019). Extensive
CRISPR RNA Modification Reveals Chemical Compatibility and Structure-Activity
Relationships for Cas9 Biochemical Activity. Nucleic Acids Res..

[4] Yin H., Song C. Q., Suresh S., Kwan S. Y., Wu Q., Walsh S., Ding J., Bogorad R. L., Zhu L. J., Wolfe S. A., Koteliansky V., Xue W., Langer R., Anderson D. G. (2018). Partial DNA-Guided Cas9 Enables Genome Editing with
Reduced off-Target Activity. Nat. Chem. Biol..

[5] Mir A., Alterman J. F., Hassler M. R., Debacker A. J., Hudgens E., Echeverria D., Brodsky M. H., Khvorova A., Watts J. K., Sontheimer E. J. (2018). Heavily
and Fully Modified RNAs Guide Efficient SpyCas9-Mediated
Genome Editing. Nat. Commun..

[6] Andrews B. I., Antia F. D., Brueggemeier S. B., Diorazio L. J., Koenig S. G., Kopach M. E., Lee H., Olbrich M., Watson A. L. (2021). Sustainability
Challenges and Opportunities in Oligonucleotide Manufacturing. J. Org. Chem..

[7] Abe A., Časar Z. (2025). Overview and
Recent Advances in the Purification and
Isolation of Therapeutic Oligonucleotides. Org.
Process Res. Dev..

[8] Pearson W. H., Berry D. A., Stoy P., Jung K.-Y., Sercel A. D. (2005). Fluorous
Affinity Purification of Oligonucleotides. J.
Org. Chem..

[9] Studzińska S., Zawadzka E., Bocian S., Szumski M. (2021). Synthesis and Application
of Stationary Phase for DNA-Affinity Chromatographic Analysis of Unmodified
and Antisense Oligonucleotide. Anal. Bioanal.
Chem..

[10] Flemmich L., Bereiter R., Micura R. (2024). Chemical Synthesis
of Modified RNA. Angew. Chem., Int. Ed..

[11] Fang S., Fueangfung S. (2010). Scalable Synthetic
Oligodeoxynucleotide Purification
with Use of a Catching by Polymerization, Washing, and Releasing Approach. Org. Lett..

[12] Fang S., Fueangfung S., Lin X., Zhang X., Mai W., Bi L., Green S. A. (2011). Synthetic
Oligodeoxynucleotide Purification by Polymerization
of Failure Sequences. Chem. Commun..

[13] Yin Y., Arneson R., Yuan Y., Fang S. (2025). Long Oligos: Direct
Chemical Synthesis of Genes with up to 1728 Nucleotides. Chem. Sci..

[14] Fang S., Bergstrom D. E. (2004). Reversible
5′-End Biotinylation and Affinity
Purification of Synthetic RNA. Tetrahedron Lett..

[15] Fang S., Bergstrom D. E. (2003). Reversible Biotinylation Phosphoramidite for 5′-End-Labeling,
Phosphorylation, and Affinity Purification of Synthetic Oligonucleotides. Bioconjugate Chem..

[16] Igata Y., Saito-Tarashima N., Matsumoto D., Sagara K., Minakawa N. A. (2017). ‘Catch
and Release’ Strategy towards HPLC-Free Purification of Synthetic
Oligonucleotides by a Combination of the Strain-Promoted Alkyne-Azide
Cycloaddition and the Photocleavage. Bioorg.
Med. Chem..

[17] He M., Wu X., Mao S., Haruehanroengra P., Khan I., Sheng J., Royzen M. (2021). Bio-Orthogonal Chemistry
Enables Solid Phase Synthesis
and HPLC and Gel-Free Purification of Long RNA Oligonucleotides. Chem. Commun..

[18] Greenberg M. M., Gilmore J. L. (1994). Cleavage of Oligonucleotides
from Solid-Phase Supports
Using o-Nitrobenzyl Photochemistry. J. Org.
Chem..

[19] Luo J., Uprety R., Naro Y., Chou C., Nguyen D. P., Chin J. W., Deiters A. (2014). Genetically Encoded Optochemical
Probes for Simultaneous Fluorescence Reporting and Light Activation
of Protein Function with Two-Photon Excitation. J. Am. Chem. Soc..

[20] Menge C., Heckel A. (2011). Coumarin-Caged DG for
Improved Wavelength-Selective
Uncaging of DNA. Org. Lett..

[21] Zhang D., Zhou C. Y., Busby K. N., Alexander S. C., Devaraj N. K. (2018). Light-Activated Control of Translation
by Enzymatic
Covalent MRNA Labeling. Angew. Chem., Int. Ed..

[22] Bollu A., Schepers H., Klöcker N., Erguven M., Lawrence-Dörner A., Rentmeister A. (2024). Visible Light Activates Coumarin-Caged MRNA for Cytoplasmic
Cap Methylation in Cells. Chem. - Eur. J..

[23] Li H., Miller M. J. (2000). Syntheses and Binding
Studies of Oligonucleotides Containing
N-Hydroxycarbamate Linkages: Potential DNA Cleaving Antisense Oligomers. Tetrahedron Lett..

[24] Yamazoe S., Liu Q., McQuade L. E., Deiters A., Chen J. K. (2014). Sequential Gene
Silencing Using Wavelength-Selective Caged Morpholino Oligonucleotides. Angew. Chem., Int. Ed..

[25] He M., Wu X., Mao S., Haruehanroengra P., Khan I., Sheng J., Royzen M. (2021). Non-Chromatographic
Purification of Synthetic RNA Using
Bio-Orthogonal Chemistry. Curr. Protoc..

[26] Eckstein F. (2014). Phosphorothioates,
Essential Components of Therapeutic Oligonucleotides. Nucleic Acid Ther..

[27] Kanavarioti A. (2019). HPLC Methods
for Purity Evaluation of Man-Made Single-Stranded RNAs. Sci. Rep..

[28] Versteegen R. M., Rossin R., Filot I. A. W., Hoeben F. J. M., van
Onzen A. H. A. M., Janssen H. M., Robillard M. S. (2024). Ortho-Functionalized
Pyridinyl-Tetrazines Break the Inverse Correlation between Click Reactivity
and Cleavage Yields in Click-to-Release Chemistry. Commun. Chem..

[29] Darko A., Wallace S., Dmitrenko O., Machovina M. M., Mehl R. A., Chin J. W., Fox J. M. (2014). Conformationally
Strained Trans-Cyclooctene with Improved Stability and Excellent Reactivity
in Tetrazine Ligation. Chem. Sci..

[30] Brzezinska J., Trzciński S., Strzelec J., Chmielewski M. K. (2023). From CPG
to Hybrid Support: Review on the Approaches in Nucleic Acids Synthesis
in Various Media. Bioorg. Chem..

[31] Nassir M., Gherardi L., Redman R. L., Jin Y., Yao F., Yang Y., Raheja N., Natarajan A., Butler D., Knouse K. W., Baran P. S. (2025). An Improved P­(V)
Thio-Oligonucleotide Synthesis Platform. Org.
Lett..

[32] Poredoš T., Trampuž M., Gornik T., Naveršnik K., Tisnikar M. S., Pirc S., Časar Z. (2024). Why and How
to Control P-Chirality in Phosphorothioated Therapeutic Oligonucleotides:
Analytical Challenges Associated with Determination of Stereochemical
Composition. Org. Process Res. Dev..

