# Supplementary material for: Coumarin-Based Photolabile Solid Support Facilitates Nonchromatographic Purification of RNA Oligonucleotides
Source: J Org Chem. 2025 Nov 10;90(46):16326–33. doi: 10.1021/acs.joc.5c01528 (PMC12645480; doi:10.1021/acs.joc.5c01528)
Supplement: Supplementary file 1 [file jo5c01528_si_001.pdf]

SUPPORTING INFORMATION

Coumarin-based photolabile solid support facilitates non-chromatographic purification of RNA oligonucleotides.

Ian McClain, Hilal Dagci, Bhoomika Pandit and Maksim Royzen*

Department of Chemistry, University at Albany, 1400 Washington Ave. Albany, NY 12222, USA

*email: mroyzen@albany.edu

**Table of Contents**	**Figure titles**	**Page**
Figure S1	Optimization of the photocleavage procedure	S2
Figure S2	HPLC spectra of **RNA 4** and **RNA5**	S3
Figure S3	NMR of compound **1**	S4
Figure S4	NMR of compound **2**	S5
Figure S5	NMR of compound **3**	S6
Figure S6	NMR of compound **5**	S7

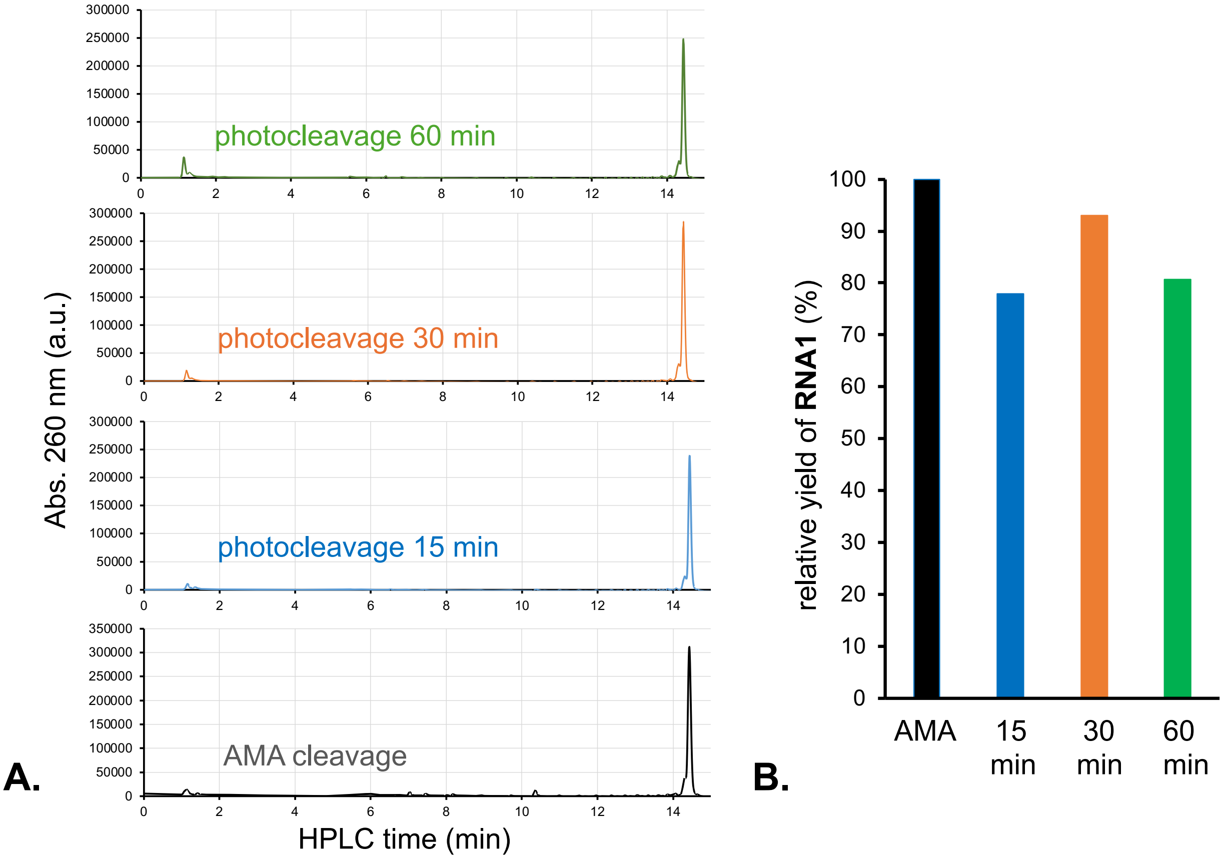

Figure S1. Optimization of the photocleavage procedure. (**A.**) HPLC analysis of **RNA1** that was cleaved from CPG either using AMA treatment or by irradiation with 456 nm light for 15, 30 or 60 min. The photocleavage was done using Kessil lamp (PR160L) at 75% power. (**B.**) Isolated yield of **RNA1** using photocleavage relative to the AMA treatment.

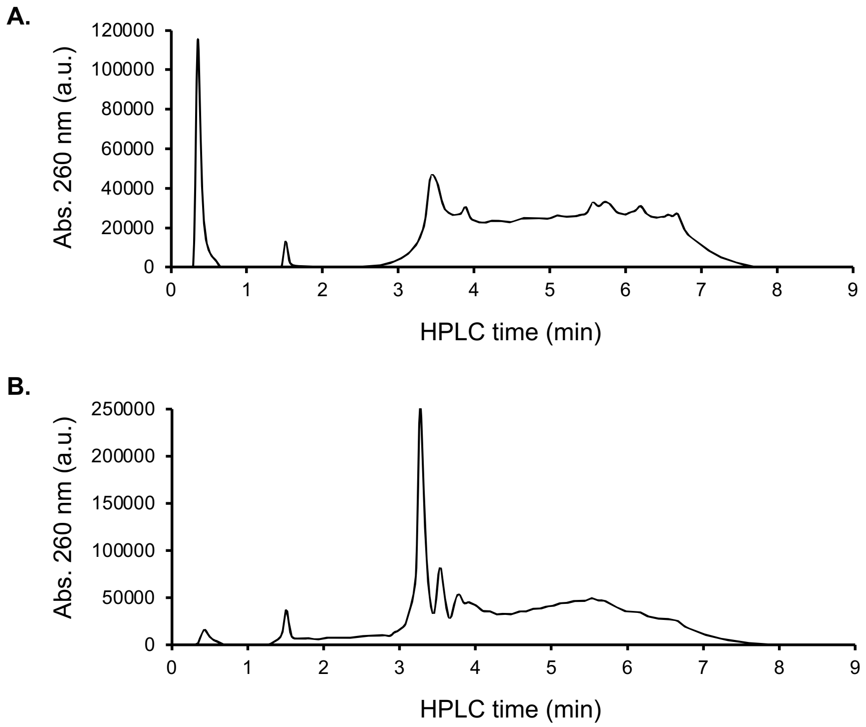

Figure S2. HPLC spectra of (**A.**) **RNA 4** and (**B.**) **RNA5**. The RNAs were synthesized using previously reported nitroaryl photolabile solid support and were isolated using the reported non-chromatographic purification method [*Chem. Commun.* **2021**, *57*, 4263].

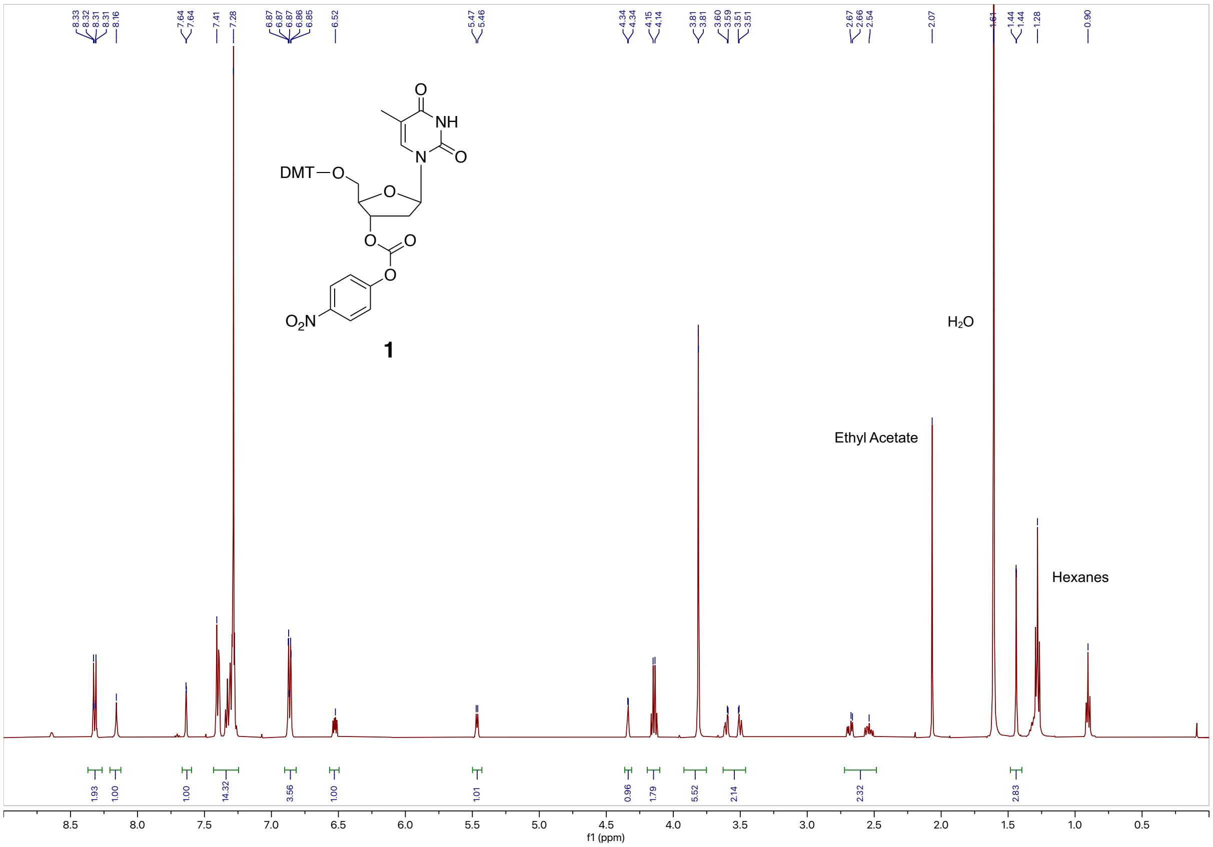

Figure S3. ^{1}H NMR (500 MHz, $CDCl_3$) of compound **1**.

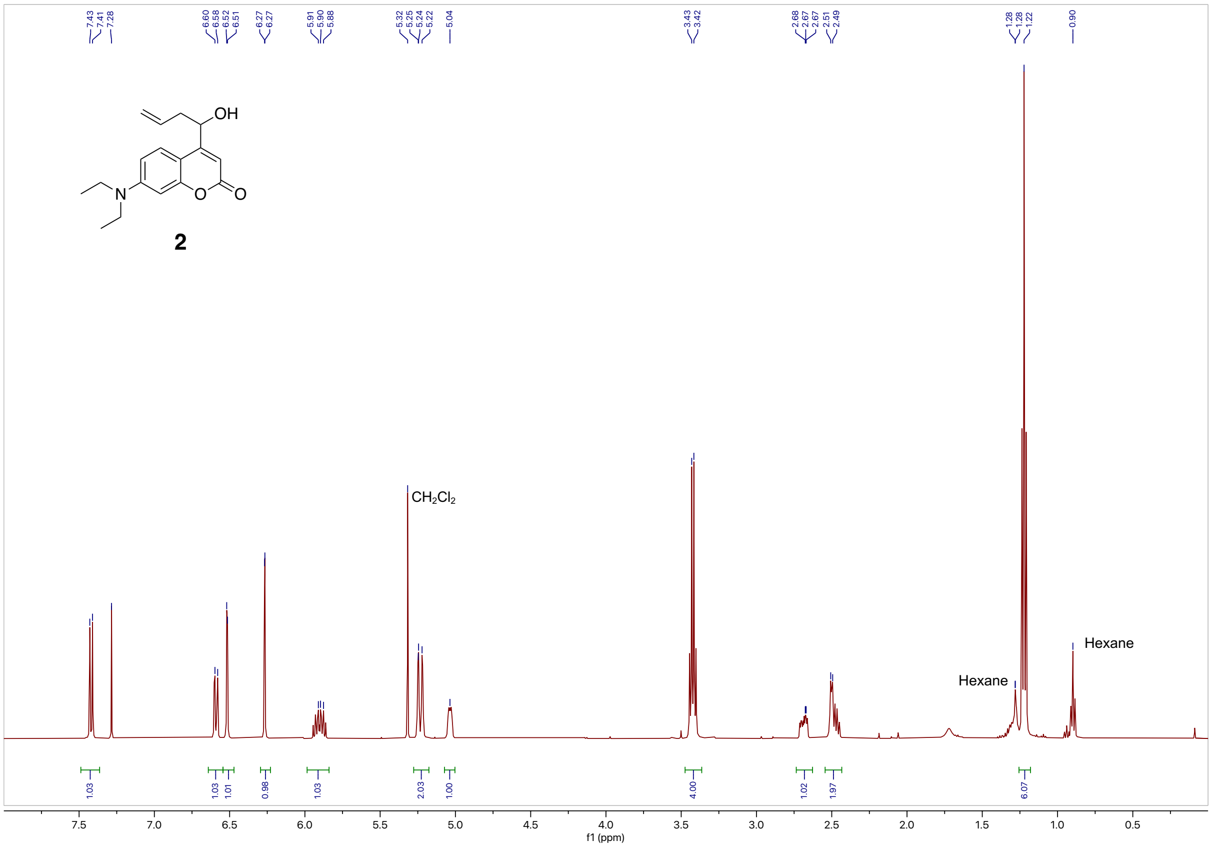

Figure S4. ^{1}H NMR (500 MHz, $CDCl_3$) of compound **2**.

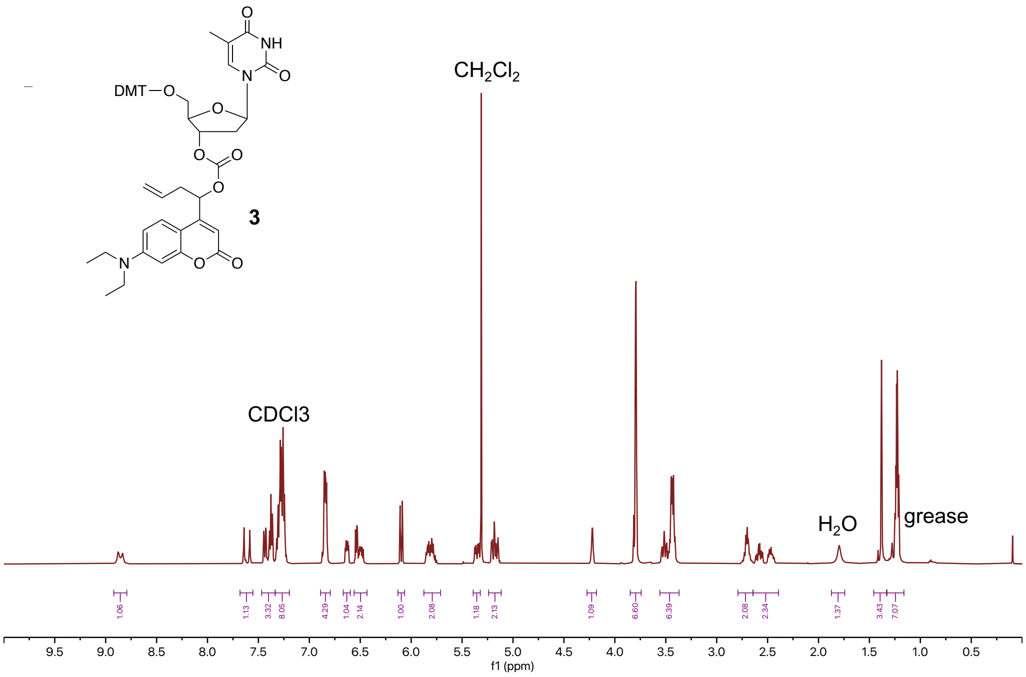

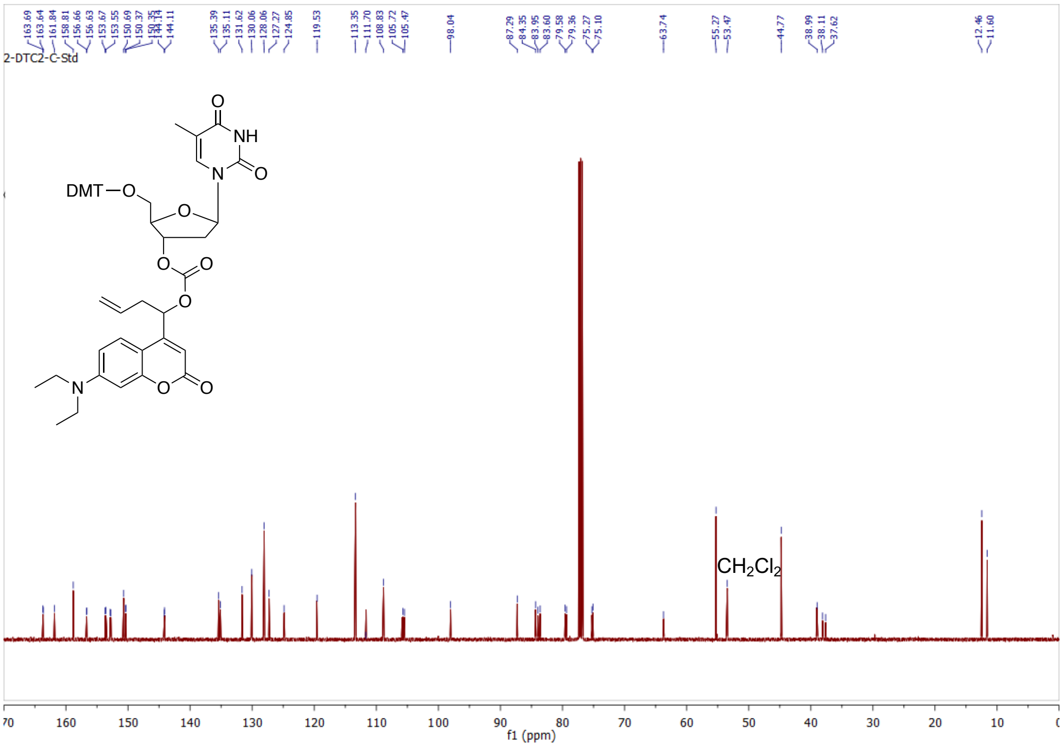

Figure S5. ^{1}H NMR (500 MHz, $CDCl_3$) and $^{13}C\{^{1}H\}$ NMR (126 MHz, $CDCl_3$) of compound **3**.

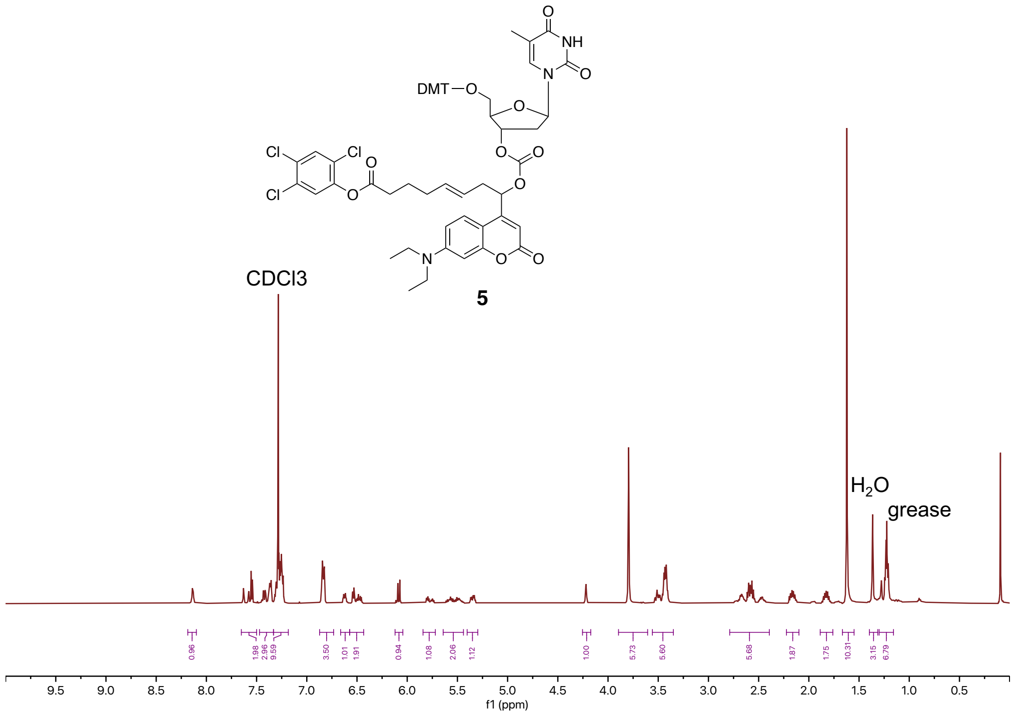

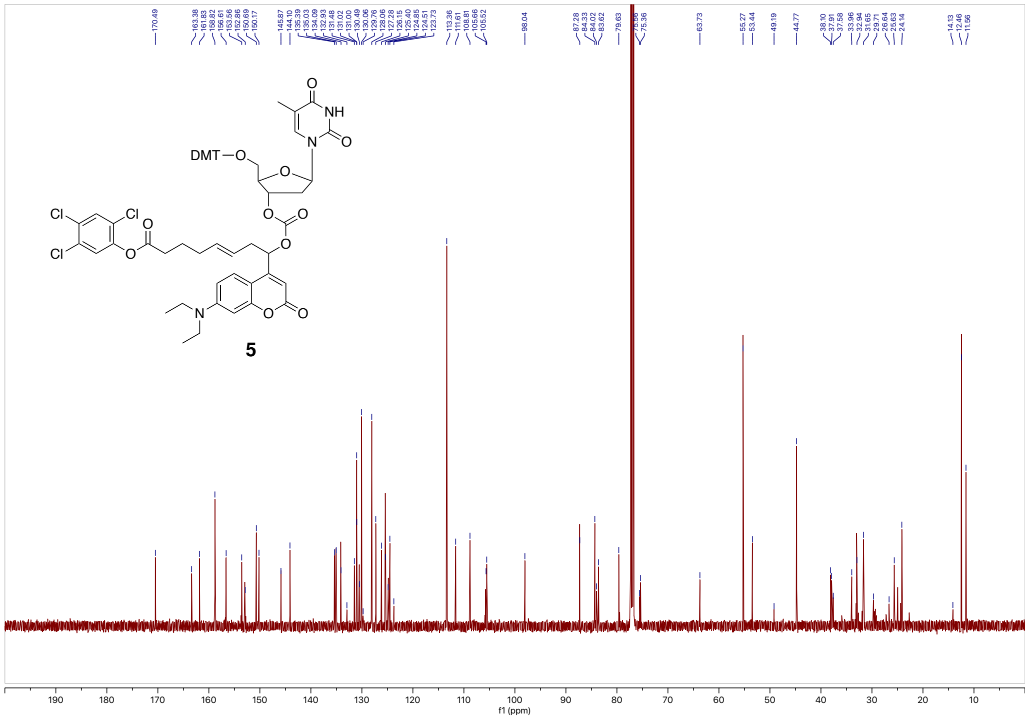

Figure S6. ^1H NMR (500 MHz, $CDCl_3$) and $^{13}C\{^1H\}$ NMR (126 MHz, $CDCl_3$) of compound **5**.
